# Supplementary material for: Localisation of corticosteroids in male mouse kidney by mass spectrometry imaging
Source: J Mol Endocrinol. 2026 Feb 20;76(2):e250099. doi: 10.1530/JME-25-0099 (PMC12927002; doi:10.1530/JME-25-0099)
Supplement: Supplementary file 1 [file supplementary_materials.pdf]

Supplementary File 1: MATLAB algorithm for calculation of signal intensity on kidney sections

```
clc
clear all
close all
load FILE_NAME_A_GirT.txt
load FILE_NAME_B_GirT.txt
load FILE_NAME_Aldo_GirT.txt
load FILE_NAME_d8B_GirT.txt
load FILE_NAME_CHCA.txt
load FILE_NAME_mz_4603264.txt
load FILE_NAME_mz_4643464.txt
load FILE_NAME_mz_4723862.txt

[intensity_a, a_coord] = compute_average_intensity( FILE_NAME_A_GirT(:,2),
FILE_NAME_A_GirT(:,5) );
[intensity_b, b_coord] = compute_average_intensity( FILE_NAME_B_GirT(:,2),
FILE_NAME_B_GirT(:,5) );
[intensity_al, al_coord] = compute_average_intensity( FILE_NAME_Aldo_GirT(:,2),
FILE_NAME_Aldo_GirT(:,5) );
[intensity_d8b, d8b_coord] = compute_average_intensity( FILE_NAME_d8B_GirT(:,2),
FILE_NAME_d8B_GirT(:,5) );
[intensity_ch, ch_coord] = compute_average_intensity( FILE_NAME_CHCA(:,2),
FILE_NAME_CHCA(:,5) );
[intensity_mz460, mz460_coord] = compute_average_intensity( FILE_NAME_mz_4603264(:,2),
FILE_NAME_mz_4603264(:,5) );
[intensity_mz464, mz464_coord] = compute_average_intensity( FILE_NAME_mz_4643464(:,2),
FILE_NAME_mz_4643464(:,5) );
[intensity_mz472, mz472_coord] = compute_average_intensity( FILE_NAME_mz_4723862(:,2),
FILE_NAME_mz_4723862(:,5) );


figure(1)
A = bar(a_coord, intensity_a);
A.FaceColor = 'b';
hold on
xline (46.5000,'--r',{'Renal pelvis'});
xline (48.1125,'--r',{'Medullary edge'});
xline (49.2375,'--r',{'Corticomedullary',' juction'});
xline (49.7625,'--r',{'Inner cortical edge'});
xline (50.8875,'--r',{'Outer cortical edge'});
ylabel('Average intesity','fontsize',16)
title('A-GirT average intensity along X-axis','fontsize',20)
set(gca,'XTick',[(46.5000+48.1125)/2 (48.1125+49.2375)/2 (49.2375+49.7625)/2
(49.7625+50.8875)/2],'XTickLabel', {'Papilla', 'Medulla', 'Inner Cortex', 'Outer Cortex' },
'FontSize', 20)
print (gcf, 'A-GirT intensity ver2.png','-dpng','-r300');


figure(2)
A = bar(b_coord, intensity_b);
A.FaceColor = 'b';
```

```
hold on
xline (46.5000,'--r',{'Renal pelvis'});
xline (48.1125,'--r',{'Medullary edge'});
xline (49.2375,'--r',{'Corticomedullary',' juction'});
xline (49.7625,'--r',{'Inner cortical edge'});
xline (50.8875,'--r',{'Outer cortical edge'});
ylabel('Average intesity','fontsize',16)
title('B-GirT average intensity along X-axis','fontsize',20)
set(gca,'XTick',[(46.5000+48.1125)/2 (48.1125+49.2375)/2 (49.2375+49.7625)/2
(49.7625+50.8875)/2],'XTickLabel', {'Papilla', 'Medulla', 'Inner Cortex', 'Outer Cortex' },
'FontSize', 20)
print (gcf, 'B-GirT intensity ver2.png','-dpng','-r300');


figure(3)
A = bar(al_coord, intensity_al);
A.FaceColor = 'b';
hold on
xline (46.5000,'--r',{'Renal pelvis'});
xline (48.1125,'--r',{'Medullary edge'});
xline (49.2375,'--r',{'Corticomedullary',' juction'});
xline (49.7625,'--r',{'Inner cortical edge'});
xline (50.8875,'--r',{'Outer cortical edge'});
ylabel('Average intesity','fontsize',16)
title('Aldo-GirT average intensity along X-axis','fontsize',20)
set(gca,'XTick',[(46.5000+48.1125)/2 (48.1125+49.2375)/2 (49.2375+49.7625)/2
(49.7625+50.8875)/2],'XTickLabel', {'Papilla', 'Medulla', 'Inner Cortex', 'Outer Cortex' },
'FontSize', 20)
print (gcf, 'Aldo-GirT intensity ver2.png','-dpng','-r300');


figure(4)
A = bar(d8b_coord, intensity_d8b);
A.FaceColor = 'b';
hold on
%xline (47.9625,'--r',{'Cortex'});
xline (46.5000,'--r',{'Renal pelvis'});
xline (48.1125,'--r',{'Medullary edge'});
xline (49.2375,'--r',{'Corticomedullary',' juction'});
xline (49.7625,'--r',{'Inner cortical edge'});
xline (50.8875,'--r',{'Outer cortical edge'});
ylabel('Average intesity','fontsize',16)
title('d8B-GirT average intensity along X-axis','fontsize',20)
set(gca,'XTick',[(46.5000+48.1125)/2 (48.1125+49.2375)/2 (49.2375+49.7625)/2
(49.7625+50.8875)/2],'XTickLabel', {'Papilla', 'Medulla', 'Inner Cortex', 'Outer Cortex' },
'FontSize', 20)
print (gcf, 'd8B-GirT intensity ver2.png','-dpng','-r300');


figure(5)
A = bar(ch_coord, intensity_ch);
A.FaceColor = 'b';
```

```
hold on
%xline (47.9625,'--r',{'Cortex'});
xline (46.5000,'--r',{'Renal pelvis'});
xline (48.1125,'--r',{'Medullary edge'});
xline (49.2375,'--r',{'Corticomedullary',' juction'});
xline (49.7625,'--r',{'Inner cortical edge'});
xline (50.8875,'--r',{'Outer cortical edge'});
ylabel('Average intesity','fontsize',16)
title('CHCA average intensity along X-axis','fontsize',20)
set(gca,'XTick',[(46.5000+48.1125)/2 (48.1125+49.2375)/2 (49.2375+49.7625)/2
(49.7625+50.8875)/2],'XTickLabel', {'Papilla', 'Medulla', 'Inner Cortex', 'Outer Cortex' },
'FontSize', 20)
print (gcf, 'CHCA intensity ver2.png','-dpng','-r300');




function [mean_intensity, unique_coord] = compute_average_intensity(coord, intensity)
    unique_coord = unique(coord);
    mean_intensity = zeros(length(unique_coord), 1);

    for i = 1:length(unique_coord)
        I = find(unique_coord(i) == coord);
        mean_intensity(i)=mean( intensity(I) )
    end
end
```

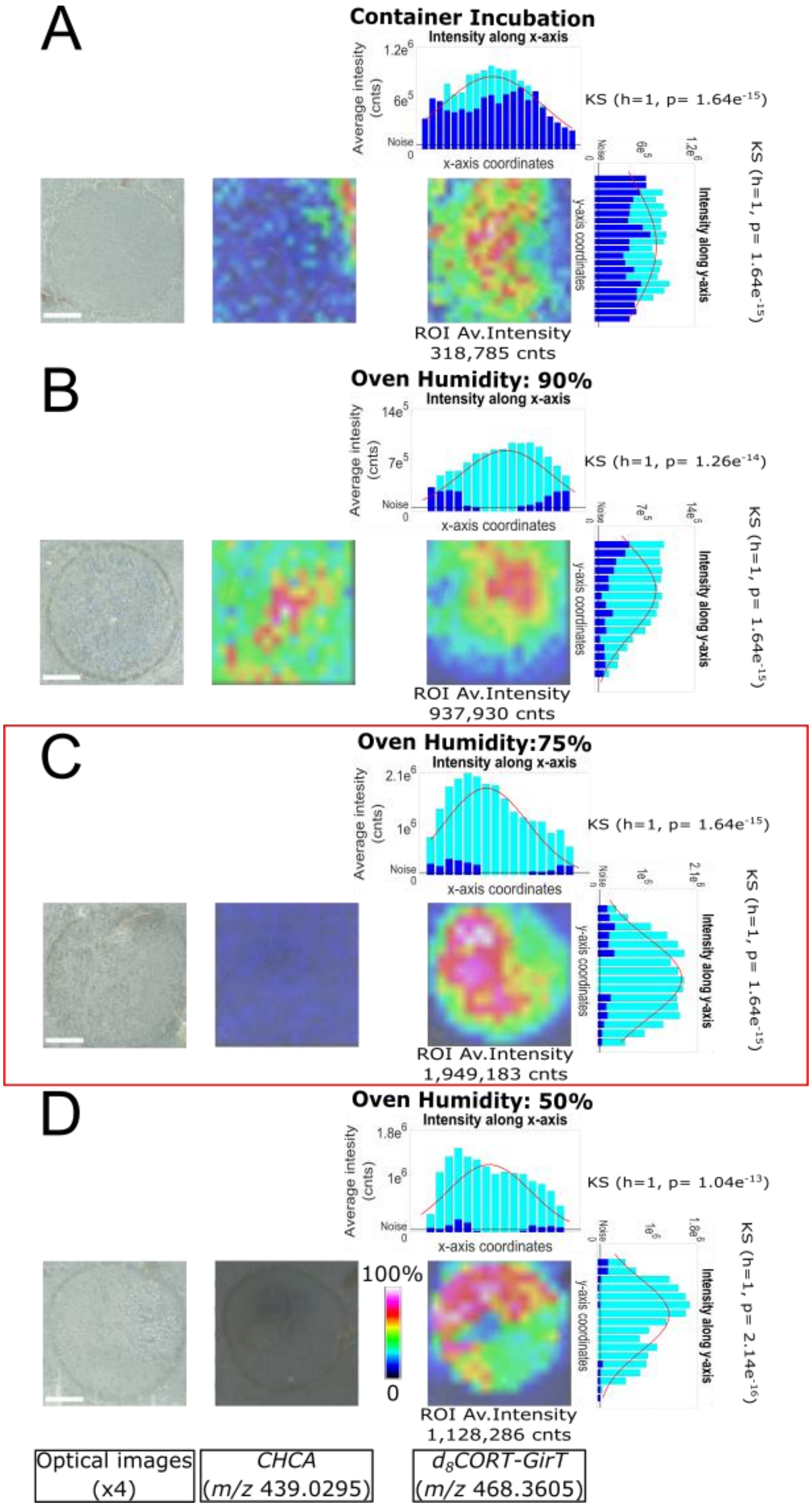
A
Container Incubation
KS (h=1, p= 1.64e-15)
ROI Av.Intensity
318,785 cnts
B
Oven Humidity: 90%
KS (h=1, p= 1.26e-14)
KS (h=1, p= 1.64e-15)
ROI Av.Intensity
937,930 cnts
C
Oven Humidity:75%
KS (h=1, p= 1.64e-15)
KS (h=1, p= 1.64e-15)
ROI Av.Intensity
1,949,183 cnts
D
Oven Humidity: 50%
KS (h=1, p= 1.04e-13)
KS (h=1, p= 2.14e-16)
100%
0
ROI Av.Intensity
1,128,286 cnts
Optical images (x4)
CHCA (m/z 439.0295)
d8CORT-GirT (m/z 468.3605)

Supplementary File 2: Method development in kidney to adapt the method of Cobice et al [13]. The main change was incubation was performed in a humidity oven under open conditions and not a sealed container. Success of derivatization was assessed by applying a spot of D_8 corticosterone standard (0.5 ng) on the surface of a cryosection of kidney. Derivatization with Girard T (GirT) and matrix application were performed and intensities and localization of the ions comprising the spot assessed using matrix assisted laser desorption ionization Fourier Transform ion cyclotron resonance mass spectrometry. Data were extracted as pixels and distributions represented in histograms, assessed for normal distribution patterns by Kolmogorov-Smirnov (KS) tests. (h=1 if the tests reject the null hypothesis that the dataset follows normal distribution at a 5% significance level, and "0" otherwise; red line=normal distribution trend line). Higher signal intensity and less diffusion was observed when humidity was 75% (C). Scale bar = 1 mm and intensity of ion signal shown against a color scale. Cnts = counts; ROI = region of interest. CHCA = α-cyano-4-hydroxycinnamic acid.

[13] D.F. Cobice, C.L. MacKay, R. Goodwin, A. McBride, P. Langridge-Smith, S.P. Webster, B.R. Walker, R. Andrew, MS imaging for dissecting steroid intracrinology within target tissues., Analytical Chemistry 85 (2013) 11576-11584.
